# Metformin promotes female germline stem cell proliferation by upregulating Gata-binding protein 2 with histone β-hydroxybutyrylation

**DOI:** 10.1186/s13287-023-03360-1

**Published:** 2023-05-26

**Authors:** Xiang Wang, Geng G. Tian, Weiwei Cheng, Xiaoli Yu, Xiaoyong Li, Ji Wu

**Affiliations:** 1 Key Laboratory of Fertility Preservation and Maintenance of Ministry of Education, School of Basic Medical Sciences, Ningxia Medical University, Yinchuan, 750004 China; 2 Key Laboratory for the Genetics of Developmental and Neuropsychiatric Disorders (Ministry of Education), Bio-X Institutes, Shanghai Jiao Tong University, Shanghai, 200240 China; 3 International Peace Maternity and Child Health Hospital, Shanghai Jiao Tong University School of Medicine, Shanghai, 200030 China

**Keywords:** Metformin, Female germline stem cells, Histone β-hydroxybutyrylation, Gata-binding protein 2

## Abstract

**Background:**

Metformin as a first-line clinical anti-diabetic agent prolongs the lifespan of model animals and promotes cell proliferation. However, the molecular mechanisms underlying the proliferative phenotype, especially in epigenetics, have rarely been reported. The aim of this study was to investigate the physiological effects of metformin on female germline stem cells (FGSCs) in vivo and in vitro, uncover β-hydroxybutyrylation epigenetic modification roles of metformin and identify the mechanism of histone H2B Lys5 β-hydroxybutyrylation (H2BK5bhb) in Gata-binding protein 2 (Gata2)-mediated proliferation promotion of FGSCs.

**Methods:**

The physiological effects of metformin were evaluated by intraperitoneal injection and histomorphology. The phenotype and mechanism studies were explored by cell counting, cell viability, cell proliferation assay and protein modification omics, transcriptomics, chromatin immunoprecipitation sequencing in FGSCs in vitro.

**Results:**

We found that metformin treatment increased the number of FGSCs, promoted follicular development in mouse ovaries and enhanced the proliferative activity of FGSCs in vitro. Quantitative omics analysis of protein modifications revealed that H2BK5bhb was increased after metformin treatment of FGSCs. In combination with H2BK5bhb chromatin immunoprecipitation and transcriptome sequencing, we found that Gata2 might be a target gene for metformin to regulate FGSC development. Subsequent experiments showed that Gata2 promoted FGSC proliferation.

**Conclusion:**

Our results provide novel mechanistic understanding of metformin in FGSCs by combining histone epigenetics and phenotypic analyses, which highlight the role of the metformin-H2BK5bhb-Gata2 pathway in cell fate determination and regulation.

**Supplementary Information:**

The online version contains supplementary material available at 10.1186/s13287-023-03360-1.

## Introduction

Metformin is a small molecule belonging to the synthetic anti-diabetic biguanide family, which is related to guanidine compound galegine extracted from French lilac (*galega officinalis*) [1, 2]. Initially, metformin was used as an ideal first-line clinical treatment for type II diabetes. In the twenty-first century, it was found to have additional functions, such as prolonging the lifespan of various animal models, anti-inflammatory and anti-oxidative stress effects, regulating protein homeostasis, anti-cancer effects, and improving the prognosis of tumor patients [3–7]. In male reproductive research, administration of metformin rescues obesity-induced abnormalities in sperm quality and motility in rats [8]. Metformin treatment also improves sperm concentration and motility and reduces abnormal sperm production in obese men [8–10]. Moreover, it regulates and improves pituitary-luteinizing hormone pulsatility and Leydig cell steroid production [9]. Metformin treatment also reduces cellular oxidative stress induced by testicular ischemia and confers reproductive protection [11]. In females, metformin alleviates the negative effects of hyperandrogenism on oocyte quality in polycystic ovarian syndrome (PCOS), restores the ovarian cycle in PCOS patients, reverses some ovulatory dysfunction caused by PCOS, reduces the risk of gestational diabetes, and improves embryonic development [12–16]. Additionally, metformin regulates stem cell development in drosophila, mice, humans, and other species through multiple pathways. It delays senescence of mesenchymal stem cells [17], prolongs the cell cycle of intestinal stem cells [18, 19], promotes self-renewal of primary neural progenitor cells [20], expands the germinal pool capacity of adult neural stem cells [21], and improves the disturbance of myelin regeneration of oligodendrocyte progenitor cells to regulate physiological functions of the body [22]. However, the developmental regulation and mechanism of metformin in germline stem cells remain unclear.

Female germline stem cells (FGSCs) are located on the ovarian cortex surface beneath the epithelium of postnatal mice and possess dual characteristics of self-renewal similar to stem cells and transmission of genetic information as germ cells [23]. Furthermore, FGSCs have been found in postnatal ovaries of rats, pigs, sheep, monkeys, and humans, indicating that FGSCs exist widely in mammals [24–32].

This finding provides a new perspective for treatment of infertility, which is of great significance for maintaining ovarian functions, improving follicular quality, and increasing the pregnancy rate of female mammals. In more than 10 years of FGSC research, systematic studies of small molecular compounds in FGSC toxicological evaluation and developmental regulation mechanisms have provided a basis for the clinical application of small molecular compounds [33–38].

In recent years, with the comprehensive application of high-sensitivity mass spectrometry and biochemical analyses, in addition to traditional lysine acetylation, methylation, and phosphorylation [39], a variety of novel acylation modifications derived from cellular metabolic intermediates have been discovered, such as succinylation, crotonylation, and lactylation [40, 41]. These discoveries greatly expand our understanding of histone epigenetic modifications. Histone Lys β-hydroxybutyrylation (Kbhb) is a novel acylation modification that has been identified in four modification sites in mice and humans [42]. Under low carbon metabolism conditions, H3K9bhb modification is enriched in the promoter regions of starvation response pathway genes, participates in the activation and regulation of related genes, and is distinct from gene categories of acetylation and methylation [42]. Furthermore, histone Kbhb is involved in epigenetic regulation of various physiological states. For example, CD8 + memory T cells express Foxo1 and PGC-1α through histone H3K9bhb modification and then cooperatively upregulate Pck1 expression. This leads to a flow of carbon along glycogen and pentose phosphate pathways, which is associated with the development of memory T cells [43]. Additionally, hepatoma cells indirectly increase the modification of histone H3K9bhb through the R-loop to promote oncogene expression, which results in a poor cancer prognosis [44]. However, the role of histone hydroxybutyrylation in reproductive development and the related mechanisms remain unclear.

In this study, we investigated the regulatory effect and mechanism of metformin on the development of FGSCs in vivo and in vitro. Our data revealed a novel histone modification pattern in the regulation of FGSC development, which contributes to functional recovery of the ovary and maintenance of fertility.

## Materials and methods

### Chemical compound

Metformin (PHR1084) purchased from Sigma-Aldrich (Louis, MO, USA) was diluted directly in PBS to various concentrations.

### Animals

Female ICR mice (12 and 28 weeks old) were purchased from SLAC Laboratory (Shanghai, China) and maintained in an auto-controlled temperature and humidity environment with 12-h/12-h light/dark cycle and ad libitum access to food and water. This study was performed in accordance with the recommendations in the Guide for the Care and Use of Laboratory Animals and relevant Chinese laws and regulations. All animal procedures were approved by the Institutional Animal Care and Use Committee (IACUC) of Shanghai Jiao Tong University. (Title: Mechanism for transdifferentiation of spermatogonia into germline stem cells and germ cells; Number: A2016084; Date: Oct 18, 2016). We declared that our manuscript reporting adheres to the ARRIVE guidelines (http://www.nc3rs.org.uk/page.asp?id=1357) for the reporting of animal experiments.

### Culture of FGSCs

The FGSC line was cultured as described previously [23, 45, 46]. In brief, FGSCs were cultured on mitotically inactivated STO (SIM mouse embryo-derived thioguanine and ouabain-resistant) feeder cells at 37 °C with 5% CO2. The culture medium was minimum essential medium alpha (12000022, Gibco), supplemented with 10% fetal bovine serum (FBS03ES-5001, Front), 10 ng/mL human basic fibroblast growth factor (10018b, PeproTech), 10 ng/mL mouse glial cell line-derived neurotrophic factor (45044, PeproTech), 10 ng/mL mouse epidermal growth factor (31,509, PeproTech), 10 ng/mL mouse leukemia inhibitory factor (sc4378, Santa Cruz Biotechnology), 1 mM non-essential amino acids (11140050, Gibco), 2 mM L-glutamine (A2916801, Gibco), 50 U/mL penicillin and 50 μg/mL streptomycin (15070063, Gibco), 1 mM pyruvate (11360070, Gibco), and 100 μM β-mercaptoethanol (M3148, Sigma-Aldrich). FGSCs were subcultured every 3 or 4 days by digestion with TrypLE™ Express (12604021, Gibco) at a 1:4 or 5 split ratio. The culture medium was changed every 2 days. FGSCs were identified by RT-PCR and immunofluorescence (Additional file 1: Fig. S1).

### Metformin treatment of mice

Female ICR mice (12 and 28 weeks old, *n* = 20 each) were allowed to acclimate for 1 week and under the premise of 4R (reduction, refinement, replacement, responsibility) principle in animal experiments. Then, mice were weighed and recorded in order of weight from lowest to highest, and four rearing rooms were assigned, with 5 mice in each rearing room. Random grouping was adopted as follows: mice were assigned according to rearing rooms from 1 to 4 and weight from lowest to highest, starting from rearing room 1 in the first round. In the second round, the mice were allocated from rearing room 2, and rearing room 1 was completed. In the third round, the mice were allocated from rearing room 3, and rearing rooms 1 and 2 were completed until the end of the four rounds. A new round was allocated from rearing room 1 in the same way as above until the mice were allocated. The experimental groups (12 and 28 weeks, *n* = 10 each) were treated with metformin intraperitoneally (i.p. 200 mg/kg) [20, 21] and the remaining mice as the control group were treated with PBS for 7 days. Then, 5-ethynyl-2ʹ-deoxyuridine (EdU, C00053, RiboBio) injection (i.p., 10 mg/kg, refer to the instruction of test kits) was performed at 1 and 3 weeks after metformin treatment (12 and 28 weeks, *n* = 5 each) to mark proliferating FGSCs. The injection procedure was operated at a sterilized workstation, and disposable syringes were used. The injection site was adequately disinfected before injection to prevent infection of the injection wound. The veterinarian was responsible for daily mental state and wound checks. The mice were euthanized by carbon dioxide at 24 h after EdU injection. Avoid the process to other mice for suffering from fear and mental pain.

### Hormone analysis

Sera were separated from collected blood samples after euthanasia, immediately. Serum levels of estradiol and progesterone were measured by an Access Immunoassay System (Beckman Coulter, UniCelDxl 800, USA) in accordance with the manufacturer’s instructions.

### Histological analyses and ovarian follicle counting

Mouse ovaries collected at the two time points were fixed with 4% paraformaldehyde, embedded in paraffin, and sectioned into 6-μm-thick slices. Sections were dewaxed, hydrated, and stained with hematoxylin and eosin (HE). Experimental procedures were performed in accordance with previous studies with some modification [23, 46]. Ovarian follicles were counted as described previously [47, 48]; in brief, the number of all types of follicles in the left and right ovarian slices of each mouse was recorded, and the repeated sections in the same position were counted only once. Finally, the total number of follicles in each mouse ovary in different treatment groups was counted and analyzed.

### Immunofluorescence of ovarian tissue

Immunofluorescence staining was performed using a Cell-Light Apollo 567 Stain Kit (C10371-1, RiboBio) in accordance with the manufacturer’s protocol. Briefly, section dewaxing and rehydration were conducted as described for HE staining, and antigen retrieval was performed in citric acid buffer (10 mM sodium citrate, pH 6.0) by microwaving. Sections were permeabilized in 0.5% Triton X-100 at room temperature for 20 min, and EdU staining was performed according to the manufacturer’s protocol. Then, 10% goat serum was applied as a blocking regent at room temperature for 30 min, followed by incubation with the primary anti-Mvh antibody at 4 °C overnight and then with a CoraLite488-conjugated secondary antibody (1:200; SA00013-2, Proteintech) at room temperature for 1 h. Nuclei were counterstained with Hoechst 33342 at room temperature for 30 min. Sections were mounted as described for HE staining. Images were captured under a Leica fluorescence microscope (Leica, DM2500, Germany).

### Cell counting

FGSCs were cultured in 24-well plates at 10^4^ cells/well overnight and then treated for 24 and 48 h with various concentrations of metformin. Then, the cells were digested, resuspended in PBS, and counted using a hemocytometer.

### Cell viability assay

FGSCs were seeded at 2000 cells/well in 96-well plates and cultured overnight at 37 °C with 5% CO2. The cells were treated with various concentrations of metformin for 24 h, and then, cell viability was measured by Cell Counting Kit-8 (CCK-8, C0038, Beyotime) in accordance with the manufacturer’s instructions. Optical density was measured at 450 nm using a microplate reader (Bio-Tek, Thermo Fisher Scientific, USA).

### Cell proliferation assay

FGSCs were cultured in 48-well plates at 5000 cells/well overnight. After treatment with various concentrations of metformin for 24 h, a Cell-Light EdU Apollo 567 in vitro kit (C10310-1, RiboBio) was used to evaluate cell proliferation in accordance with the manufacturer’s protocol. The cells were incubated with 50 μM EDU for 2 h, fixed with 4% paraformaldehyde for 30 min at room temperature, neutralized in 2 mg/mL glycine for 5 min, and incubated for 10 min on a shaking table in PBS containing 0.5% Triton X-100) for permeabilization. Then, the cells were treated with 1 × Apollo staining solution for 30 min, followed by washing with PBS containing 0.5% Triton X-100 three times. And 1 × Hoechst 33342 solution was used for counterstaining. Finally, an inverted fluorescence microscope (Leica, DMI3000B, Germany) was used to capture images that were analyzed by ImageJ. In brief, three visual fields were taken from each well in each treatment group, and the red fluorescence (EDU) and blue fluorescence (Hoechst 33,342) in each visual field were counted. The cell proliferation index was determined as the ratio of EDU to Hoechst 33342.

### RT-PCR and real-time qRT-PCR

Total RNA was extracted from FGSCs using Trizol reagent (15596026, Life Technologies). The RNA concentration was measured using a Nano Drop 2000 spectrophotometer (Thermo Fisher Scientific, USA). Approximately 1000 ng total RNA was reverse-transcribed into cDNA using a reverse transcription kit (11141ES60, YEASEN). RT-PCR was performed with 2 × HieffPCR Master Mix (10102ES03, YEASEN) in a Master cycler PCR (Eppendorf AG, 6331, Germany) using touchdown PCR mode (95 °C for 5 min, followed by 20 cycles at 95 °C for 30 s, touchdown annealing at 65–50 °C, 72 °C for 30 s, and then 15 cycles at 95 °C for 30 s, 50 °C for 30 s, 72 °C for 30 s, and final extension at 72 °C for 10 min). RT-PCR products were separated on 1.5% agarose gels and imaged with an AlphaImager EP bioimaging system (Alpha Innotech, USA). Real-time qRT-PCR analysis was carried out with a HifairIII One Step RT-qPCR SYBR Green Kit (11184ES08, YEASEN) in an Applied Biosystems Real-Time PCR System (Thermo Fisher Scientific, 7500, USA) with the following reaction conditions: 50 °C for 2 min, 95 °C for 10 min, and then 40 cycles of 95 °C for 15 s and 60 °C for 60 s. Data were analyzed by the 2-ΔΔCt method. Primers are shown in Additional file 6: Table S1 and Additional file 7: Table S2.

### Western blotting

Cells were lysed in RIPA buffer (P0013B, Beyotime) containing InStabTMProtease Cocktail inhibitor (20123ES10, YEASEN), scraped into an EP tube using a BeyoGold cell scraper (FSCP023, Beyotime), and incubated for 5 min after 20 s of vortexing for six times in total. The whole procedure was conducted on ice. Protein concentrations were measured by a BCA protein quantification kit (YEASEN, 20201ES76). A total of 20–40 µg proteins was separated using a 12.5% SDS-PAGE gel fast preparation kit (PG113, Epizyme) and then transferred to Immobilon-P PVDF membranes (Millipore, IPVH00010, Merk, Germany). Membranes were blocked with 5% dry non-fat milk in Tris-buffered saline Tween 20 (TBST) for 1.5 h with gentle shaking at room temperature and then incubated at 4 °C overnight (16–18 h) in QuickBlock™ primary antibody dilution buffer (P0256, Beyotime) with the following primary antibodies: anti-Kbhb (1:2000; PTM-1201, PTM), anti-H2BK5bhb (1:2000; PTM-1230, PTM), anti-histone H3 (1:2000; 17168-1-AP, Proteintech), anti-Gata2 (1:2000; ab109241, Abcam, UK), or anti-GAPDH (1:50000; 60004-1-Ig, Proteintech). The membranes were washed with Tris-buffered saline Tween 20 three times for 10 min each, incubated for 2 h with secondary antibodies (1:2000; SA00001-1 or SA00001-2, Proteintech) diluted in blocking buffer, and then washed three times with TBST for 10 min each. Finally, protein brands were visualized using ECL reagent (MA0186, Meilunbio) and scanned by a Gel Imager System (Tanon, 4600SF, China). The density of protein bands was calculated by ImageJ.

### Immunofluorescence

FGSCs were cultured to 80% confluence in 24-well plates. Then, the cells were fixed in 4% paraformaldehyde for 30 min at room temperature and washed with PBS three times. Samples were blocked in 10% goat serum at room temperature for 1 h, incubated with an anti-Mvh primary antibody (1:200; ab13840, Abcam) at 4 °C overnight (16–18 h), washed with PBS three times, and then incubated with a CoraLite594-conjugated secondary antibody (1:200; SA00013-4, Proteintech) for 1 h at room temperature. Nuclei was counterstained with 4′,6-diamidino-2-phenylindole (DAPI) for 3 min. After three washes, the cells were mounted in antifade mounting medium (P0126, Beyotime) to capture images under a fluorescence microscope (Leica, DMI3000B, Germany).

### Sequencing of proteomic pan-β-hydroxybutyrylation

Protein extraction, fragmentation, enrichment, mass spectrometry, and sequencing analysis were performed by PTM Bio Company. Briefly, samples were mixed with pyrolysis buffer for ultrasonication. After centrifugation, the supernatant was collected to determine the protein concentration using the BCA kit and then subjected to trypsin digestion overnight, following the manufacturer’s instructions. After enzymatic hydrolysis, the peptides were dissolved in IP buffer and transferred to prewashed β-hydroxybutyrylated resin (PTM Bio, China) for incubation overnight. Then, the resin-bound peptides were washed and eluted three times, collected for desalinization in accordance with the C18 Zip Tip instructions and subjected to HPLC-MS/MS analysis.

### Chromatin immunoprecipitation sequencing and qPCR

Chromatin immunoprecipitation (ChIP) and input DNA fragment preparation were conducted following a published protocol (Chapter 17) [49]. In brief, cells were cross-linked with 1% formaldehyde for 8 min. The reaction was terminated by incubation in 125 mM glycine for 5 min at room temperature. Then, the cells were resuspended in lysis buffer (50 mM Tris-HCL, pH 8.0, 10 mM EDTA, pH 8.0, 1% SDS, 1 mM PMSF, 20 mM sodium butyrate, and 1 × protease inhibitors) and sheared with a BioruptorPico sonication device (Diagenode, Belgium) to 200–500 bp. Premixed Protein A + G Magnetic beads (Thermo Fisher Scientific, USA) were bound to 8–10 μg anti-H2BK5bhb at 4 °C for 2–4 h and then incubated with DNA fragment at 4 °C for 3 h or overnight for immunoprecipitation in the IP group. The input group stored at 4 °C. Finally, the DNA fragments were washed, isolated, and purified by relevant buffers and reagents. End repair and ligation with adapters were carried out using an NEBNext Ultra EndRepair/dA-Tailing Module (E7442, NEB) and NEBNext Ultra Ligation Module (E7445, NEB), respectively. High-throughput sequencing of ChIP fragments was performed using an Illumina NextSeq 500, following the manufacturer’s protocol after quality evaluation of ultrasonic fragment (Additional file 2: Fig. S2). To align the reads to the mm9 reference genome, bowtie2 was used. PCR duplicates were removed using SAMtools (version 2.0.1). Normalized genome coverage tracks were generated from uniquely mapped reads using deepTools2 (version 3.1).

DNA fragment preparation was performed as described in 2.12. ChIP-qPCR primers for Gata2, Psen2, and Ptgr2 promoters are shown in Additional file 8: Table S3. The data were normalized to the adjusted input sample and analyzed by the percent input method.

### RNA extraction and sequencing

Total RNA was extracted from FGSCs using Trizol reagent (15596026, Life Technologies). cDNA libraries were constructed using a VAHTSTM mRNA seq v2 Library Prep Kit for Illumina1 (Vanzyme, China). Sequencing libraries were constructed in accordance with the manufacturer’s protocol. The library quality was determined by a Bioanalyzer 2100 (Agilent, Santa Clara, CA, USA). The Illumina HiSeq 2500 platform (Illumina, San Diego, CA, USA) was used for RNA-sequencing (RNA-seq). The quality of RNA-seq data was evaluated using FastQC (Additional file 3: Fig. S3).

### Gene ontology and Kyoto encyclopedia of gene and genomic pathway analysis

Gene Ontology (GO) analysis was performed to identify the biochemical processes of differentially expressed mRNAs. Kyoto Encyclopedia of Genes and Genomes (KEGG) pathway analysis was applied to identify significant pathways of differentially expressed mRNAs using DAVID (http://david.abcc.ncifcrf.gov/home.jsp). Fisher’s exact test was used to identify significant results, and the false discovery rate was applied to correct *P*-values (*P* < 0.05).

### Infection by shRNA-carrying lentiviruses

FGSCs were cultured to 50% confluence in a 48-well plate and incubated with a 100-μL mixture of culture medium and lentivirus solution (1:1 volume ratio). After 24 h of infection, the cells were cultured in normal FGSC medium for 24–48 h. Then, the cells were selected with puromycin (5 µg/mL) for 5–7 days to obtain stable Gata2-knockdown FGSCs.

### Statistical analyses

All experiments were performed at least three times. Data are expressed as means ± SD. Two-tailed Student’s t-test was used to analyze differences. Statistical analysis was performed by GraphPad Prism 8.0 software (San Diego, USA). *P* < 0.05 was considered statistically significant.

## Results

### Metformin increases the number of FGSCs in mouse ovaries and promotes follicular development and luteogenesis

**Fig. 1:**
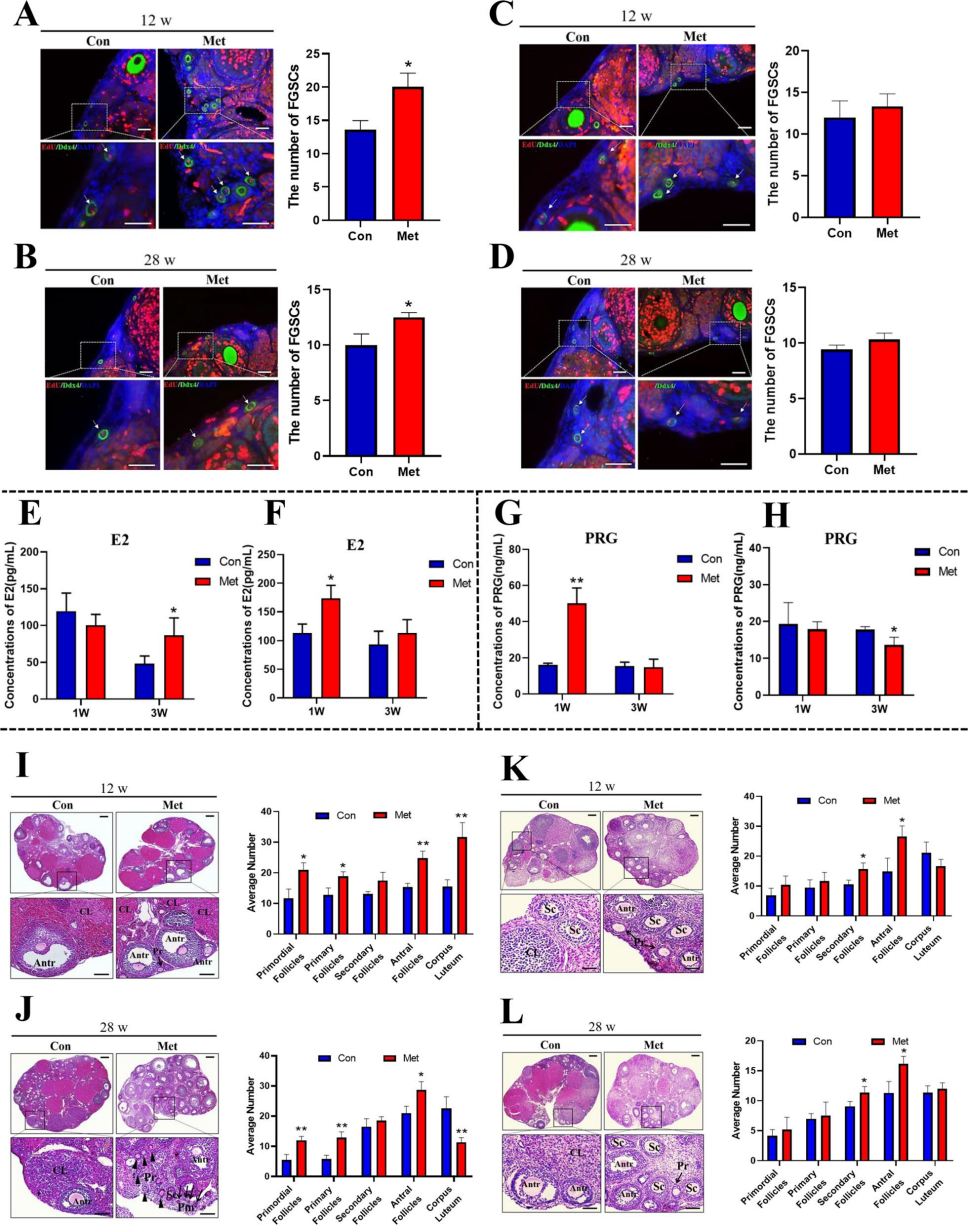
Regulatory effect of metformin on ovarian FGSC and ovarian function in mice. Figure 1–1. Metformin increases the number of FGSCs in mice ovaries. **A** Left: Dual fluorescence staining of mouse ovarian FGSCs in 12-week-old mice after metformin treatment for 1 week. Right: Statistical analysis of the number of Mvh (green) and EdU (red) double-positive cells. **B** Left: Dual fluorescence staining of mouse ovarian FGSCs in 28-week-old mice after metformin treatment for 1 week. Right: Statistical analysis of the number of Mvh (green) and EdU (red) double-positive cells. **C** Left: Dual fluorescence staining of mouse ovarian FGSCs in 12-week-old mice after metformin treatment for 3 weeks. Right: Statistical analysis of the number of Mvh (green) and EdU (red) double-positive cells. **D** Left: Dual fluorescence staining of mouse ovarian FGSCs in 28-week-old mice after metformin treatment for 3 weeks. Right: Statistical analysis of the number of Mvh (green) and EdU (red) double-positive cells. Scale bars: 50 μm. Figure 1–2. Metformin regulates the secretion of ovarian hormones in mice. **E** Changes of the serum E2 level in 12-week-old mice treated with metformin. **F** Changes of the serum E2 level in 28-week-old mice treated with metformin. **G** Changes of the serum PRG level in 12-week-old mice treated with metformin. **H** Changes of the serum PRG level in 28-week-old mice treated with metformin. Figure 1–3. Metformin regulates the development of ovarian follicles in mice. **I** Left: Histological morphology of 12-week-old mouse ovaries after metformin treatment for 1 week. Right: Statistical analysis of the number of developmental follicles in 12-week-old mice. **J** Left: Histological morphology of 28-week-old mouse ovaries after metformin treatment for 1 week. Right: Statistical analysis of the number of developmental follicles in 28-week-old mice. **K** Left: Histological morphology of 12-week-old mouse ovaries after metformin treatment for 3 weeks. Right: Statistical analysis of the number of developmental follicles in 12-week-old mice. **L** Left: Histological morphology of 28-week-old mouse ovaries after metformin treatment for 3 weeks. Right: Statistical analysis of the number of developmental follicles in 28-week-old mice. Scale bars: 100 μm. All data are presented as means ± SD. *p < 0.05, **p < 0.01 compared with the control by two-tailed Student’s t-test or one-way ANOVA and the multiple comparison test. Con: control, Met: metformin. w: week. E2: estradiol, PRG: progesterone. Pm: primordial follicle (arrows), Pr: primary follicle (arrowheads), Sc: secondary follicle, Antr: antral follicle, CL: corpus luteum

To explore the role of metformin in FGSC development in mouse ovaries, we investigated the effect of metformin on the FGSC number and ovarian functions at various time points. The results showed that the number of FGSCs in ovaries was significantly increased after treatment with metformin for 1 week compared with the control group (Fig. 1A, B). However, the difference was not statistically significant after treatment for 3 weeks (Fig. 1C, D). These data suggested that metformin promoted FGSC proliferation in mouse ovaries after 1 week of treatment, but the difference was not significant after 3 weeks of treatment.

We further evaluated the effect of metformin on the FGSC microenvironment in ovaries. First, we determined estradiol and progesterone levels in serum samples. The estradiol level in 12-week-old female mice was significantly (P < 0.05) increased after treatment for 3 weeks (Fig. 1E). The estradiol level in 28-week-old female mice was significantly different with that of treatment for 1 week (Fig. 1F). The progesterone level in 12-week-old female mice was significantly increased after treatment for 1 week compared with the control (Fig. 1G). However, the progesterone level in 28-week-old females was significantly decreased after treatment for 3 weeks (Fig. 1H). Next, we performed HE staining on collected mouse ovaries to observe structural changes. The results indicated that the proportion of follicles at various stages was significantly different after treatment for 1 or 3 weeks. After metformin treatment for 1 week, the numbers of primordial, primary, and antral follicles were increased significantly in the ovaries of both 12-week-old and 28-week-old mice. However, the number of corpus luteum was increased in 12-week-old mice, but decreased in 28-week-old mice (Fig. 1I, J). Furthermore, metformin treatment for 3 weeks significantly increased the number of secondary and antral follicles in the ovaries of both 12- and 28-week-old mice (Fig. 1K, L). These results indicated that metformin increased the number of FGSCs in mouse ovaries, regulated the secretion of reproductive hormones, promoted follicular development and luteinization, and improved the ovarian reserve capacity.

### *Metformin enhances FGSC proliferation *in vitro

**Fig. 2:**
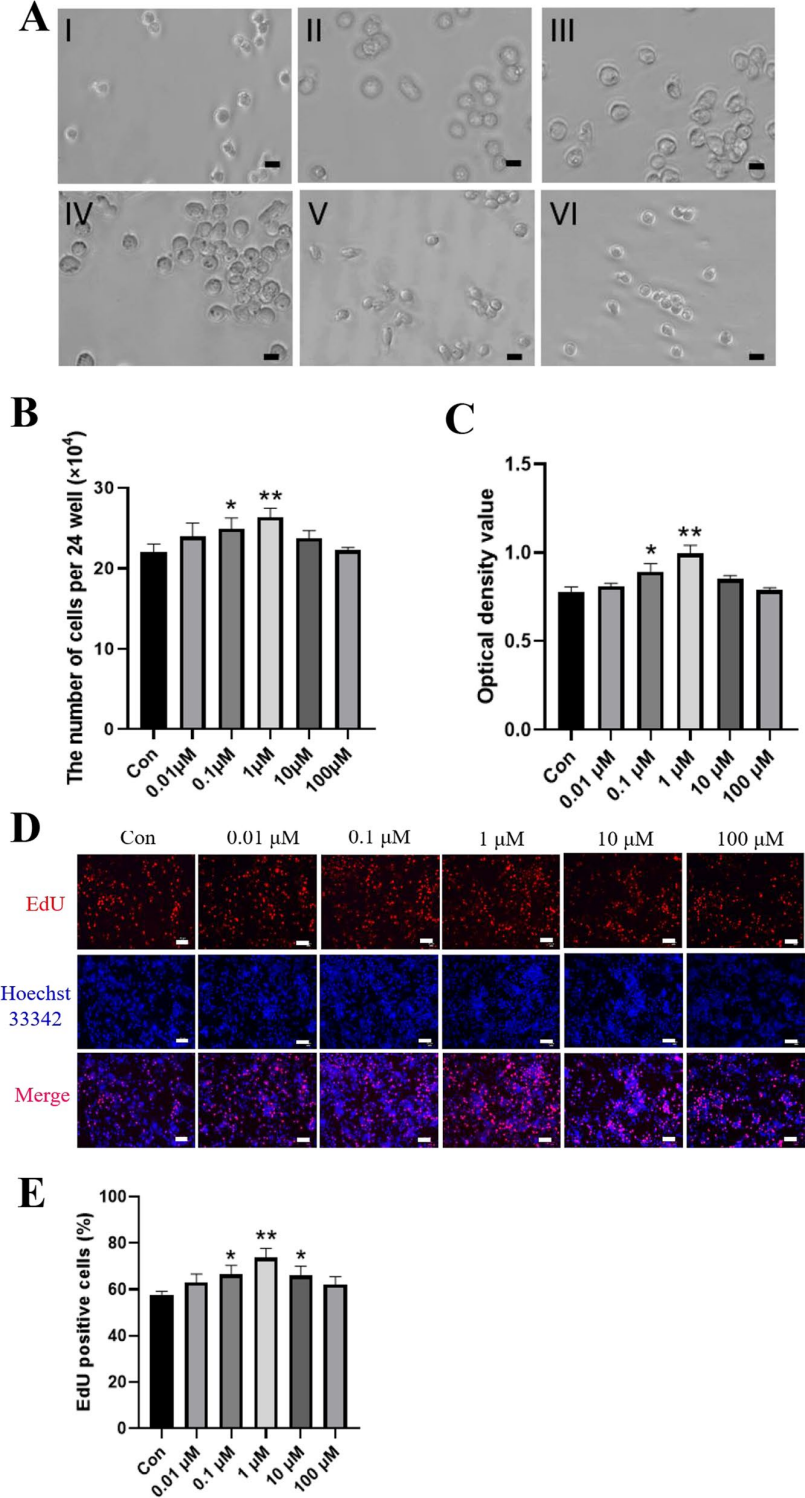
Metformin promotes FGSC proliferation in vitro. **A** Cellular morphology of FGSCs treated with various concentrations (I: control, II: 0.01 μM, III: 0.1 mM, IV: 1 μM, V: 10 μM, VI: 100 μM) of metformin (Scale bars: 20 µm). **B** Cells were counted after metformin treatment at various concentrations. **C** Cell viability assay after treatment with various metformin concentrations. **D, E** Cell proliferation after metformin treatment at various concentrations. (EdU, red; Hoechst 33,342, blue). (Scale bars: 20 μm). Right: Statistical analysis of EdU-positive cells. All data are presented as means ± SD of three biological replicates. *p < 0.05, **p < 0.01 compared with the control by one-way ANOVA and the multiple comparison test. Con: control

Considering the proliferation induction effect of metformin on mouse ovarian FGSCs, we verified this effect of metformin on FGSCs in vitro. Cultured FGSCs were characterized by identifying marker genes of germ cells: Oct4 (also known as Pou5f1, POU domain, class 5, transcription factor 1), Mvh [also known as Ddx4, DEAD (Asp-Glu-Ala-Asp) box polypeptide 4], Stella (also known as Dppa3, Developmental pluripotency-associated 3), Fragilis (also known as Ifitm3, Interferon-induced transmembrane protein 3), Dazl (deleted in azoospermia-like), and Blimp1 (also known as prdm1, PR domain-containing 1). Oct4, Mvh, Stella, Fragilis, Dazl, and Blimp1 expression was confirmed by RT-PCR (Additional file 1: Fig. S1A). Uncropped full-length agarose gels can be found in Additional file 5: Fig. S5E–G. The cell morphology was normal, and MVH positivity was further identified by immunofluorescence analysis (Additional file 1: Fig. S1B, C). These results suggested that the FGSCs had the characteristics of germline stem cells. Next, the effects of metformin on the viability and proliferative capacity of FGSCs were determined by cell counting, and CCK-8 and EdU assays. Cell counting showed that the number of cells in 0.1 and 1 μM treatment groups was significantly higher than that in the control group (Fig. 2A, B). The CCK-8 cell viability assay also showed that FGSCs treated with metformin had significantly increased cell viability compared with untreated cells (Fig. 2C). The number of EdU-positive cells (red) relative to DAPI-positive cells (Blue) calculated by ImageJ software was significantly higher in treated groups than in the control group (Fig. 2D, E). These results demonstrated that metformin treatment exerted a positive effect on FGSC proliferation.

### Metformin leads to global protein β-hydroxybutyrylation changes in FGSCs

**Fig. 3:**
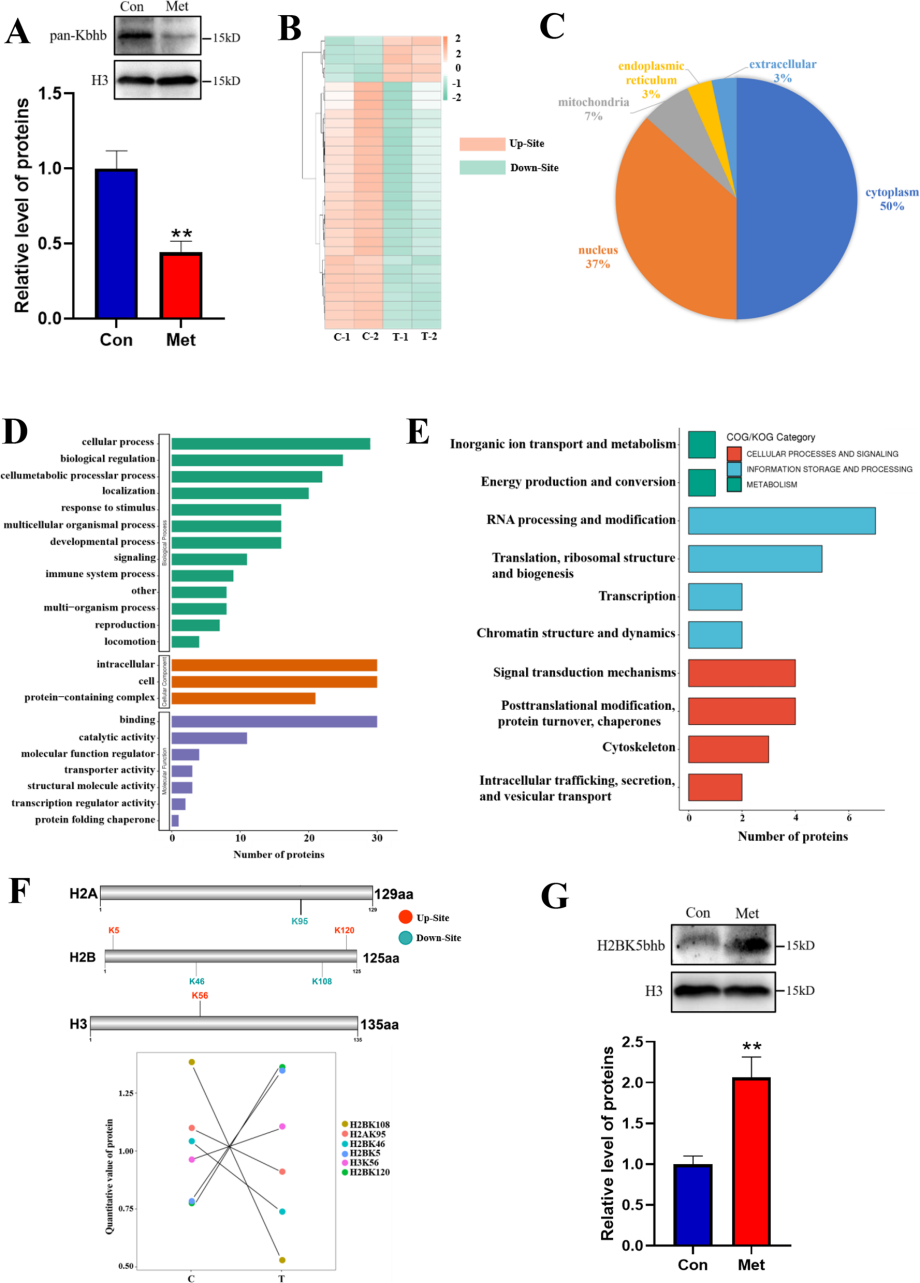
Metformin regulates the modification of total protein β-hydroxybutyrylation in FGSCs. **A** Top: Changes of Kbhb in the histone region of FGSCs treated with metformin as determined by western blotting. Bottom: Statistical analysis of western blots. Uncropped full-length gel blots can be found in Additional file 5: Fig. S5A. **B** Overview of the number of differentially expressed proteins and modification sites. **C** Proportion of subcellular localization of differentially expressed proteins modified by Kbhb. **D** GO annotation of differentially expressed proteins. **E** Homology clustering annotation of differentially expressed proteins. **F** Changes of core histone modifications in histone Kbhb. **G** Top: Western blot validation of histone H2BK5bhb modification. Bottom: Statistical analysis of western blots. Uncropped full-length gel blots can be found in Additional file 5: Fig. S5B. Western blot data are presented as mean s ± SD of three biological replicates. **p < 0.01 compared with the control by two-tailed Student’s t-test. Con: control. C-1, C-2: control repeat-1, repeat-2; Met: metformin. T-1, T-2: metformin repeat-1, repeat-2

To further reveal the mechanism of metformin affecting FGSC development, we detected whole protein Kbhb in FGSCs after metformin treatment by western blotting. The results indicated that metformin treatment significant decreased histone Kbhb in FGSCs (Fig. 3A). Uncropped full-length gel blots can be found in Additional file 5: Fig. S5A. To identify the specific proteins and sites of histone Kbhb, we enriched Kbhb-modified peptides from metformin-treated FGSCs and controls for MS analysis. The sequencing data revealed 32 modified sites of Kbhb across 30 proteins (Fig. 3B). Examining their subcellular distribution revealed a widespread cellular effect, because the subcellular localization of differentially expressed Kbhb-modified proteins was mainly distributed in the cytoplasm (50%) and partly in the nucleus (37%) (Fig. 3C). To understand the potential regulation of FGSC development by metformin-induced Kbhb, we performed Gene Ontology (GO) analysis. The results showed that many biological processes were enriched with the Kbhb modification, such as biological regulation, metabolism, reproduction and development, and molecular functions including binding or catalytic activity and transcriptional regulation activity (Fig. 3D). COG/KOG analysis identified clusters of homologous proteins, consisting of energy production and transformation, RNA processing and modification, post-translational modification, intracellular transport secretion, and vesicle transport (Fig. 3E).

Among all 32 modified sites of Kbhb, six belong to nucleosome core histone subunits, namely H2BK120, H2BK5, H3K56, H2AK95, H2BK108, and H2BK46 (Fig. 3F). Three of them were upregulated (H2BK120, H2BK5, and H3K56) and three were downregulated (H2AK95, H2BK108, and H2BK46) (Fig. 3F). Furthermore, western blotting confirmed that metformin treatment significantly promoted the upregulation of the H2BK5bhb modification in FGSCs (Fig. 3G). Uncropped full-length gel blots can be found in Additional file 5: Fig. S5B.

### Identification of potential downstream targets of histone H2BK5bhb

**Fig. 4:**
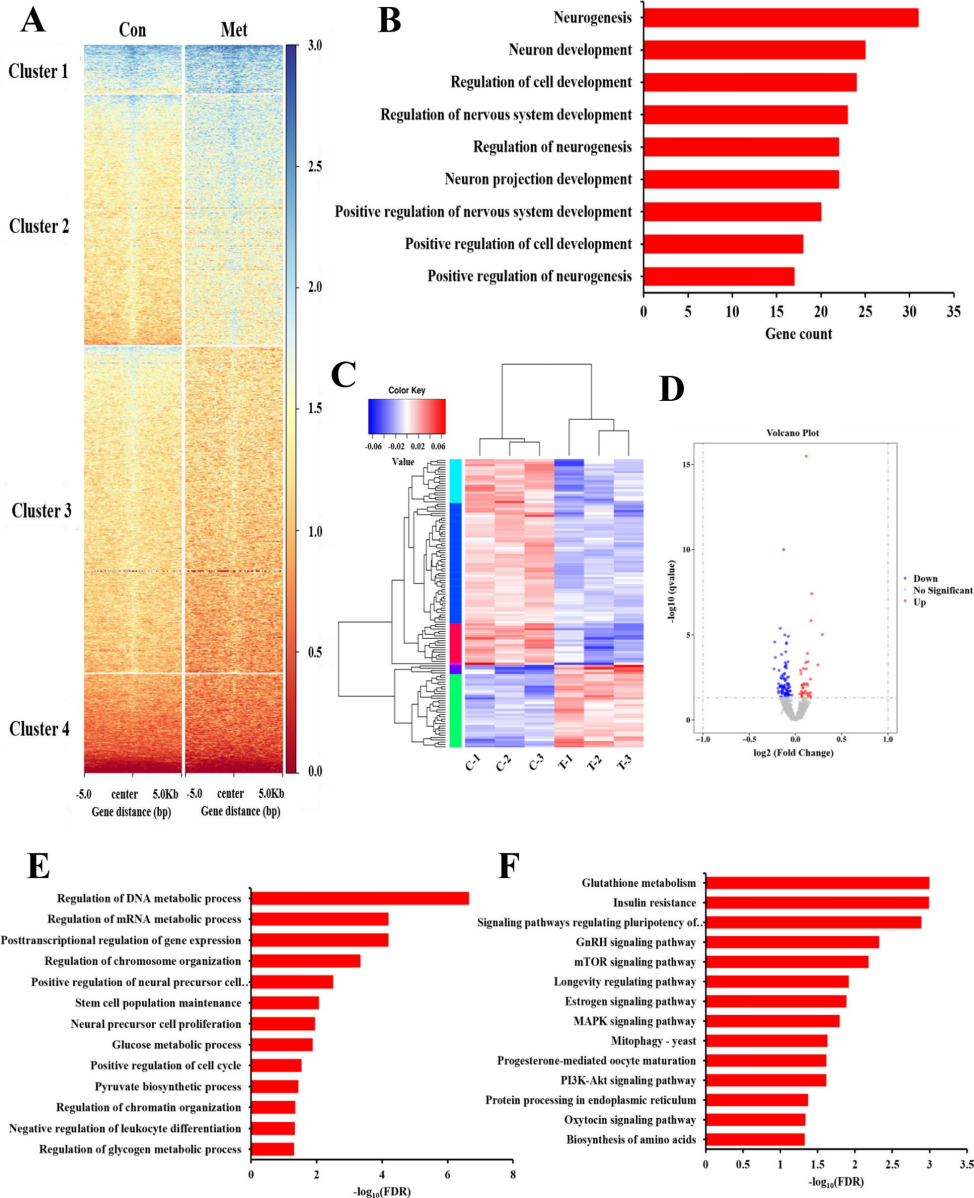
Histone H2BK5bhb Chip-seq and RNA-seq analysis of FGSCs treated with metformin. **A** Clustering of H2BK5bhb Chip-seq signals in control and treated groups. **B** GO annotation of differentially expressed genes with H2BK5bhb modification. **C** Cluster heat map of differentially expressed genes. **D** Volcano map of differentially expressed genes. **E** GO annotation analysis of RNA-seq data. **F** KEGG pathway analysis of RNA-seq data. C-1, C-2, C-3: control repeat-1, repeat-2, repeat-3; T-1, T-2, T-3: metformin repeat-1, repeat-2, repeat-3

To uncover the regulatory role of H2BK5bhb in gene expression of FGSCs after metformin treatment, we performed H2BK5bhb chromatin immunoprecipitation sequencing (ChIP-seq) in metformin-treated FGSCs versus control FGSCs. ChIP-seq data showed that differentially expressed genes could be divided into four clusters by changes in the H2BK5bhb modification (Fig. 4A). Further GO classification analysis of H2BK5bhb-specific genes revealed biological processes such as regulation of cell development and positive regulation of cell development (Fig. 4B), suggesting a regulatory effect of H2BK5bhb in mediating cell developmental events. Moreover, differentially expressed gene screening and an overview of RNA-seq data confirmed that metformin-treated FGSCs had 36 upregulated genes and 89 downregulated genes (Fig. 4C, D). Among them, GO annotation indicated that metformin treatment affected biological processes such as stem cell population maintenance and positive regulation of the cell cycle (Fig. 4E). We further analyzed enrichment of KEGG pathways and found that the enriched pathways included mTOR, estrogen, MAPK, and PI3K-Akt signaling pathways, indicating that metformin regulated the development of FGSCs through multiple pathways (Fig. 4F).

**Fig. 5:**
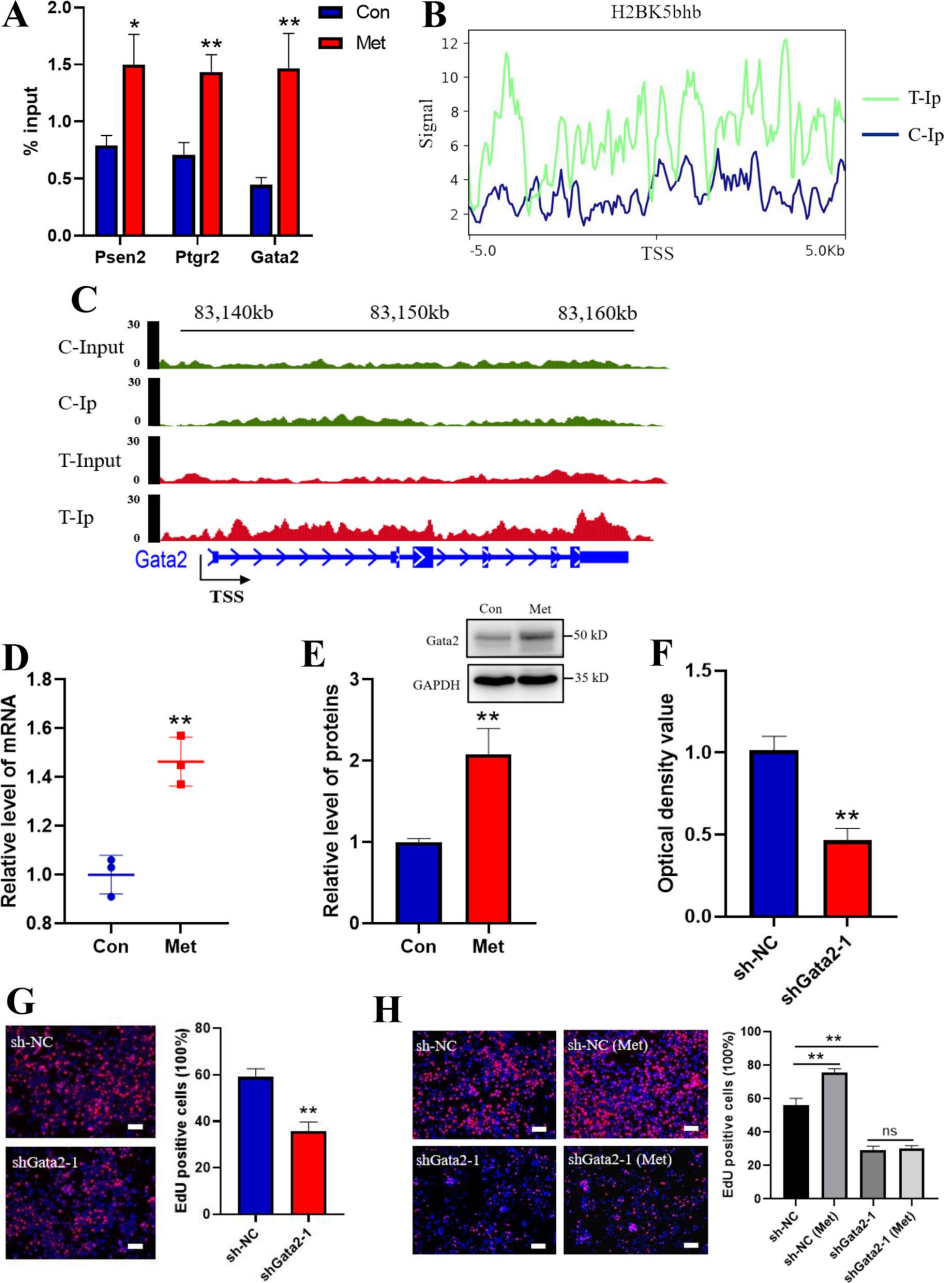
Histone β-hydroxybutyrylation activates transcription of Gata2. **A** Screening and validation of differentially expressed genes by Chip-seq. **B** Distribution of H2BK5bhb sites relative to the transcription start site (TSS). **C** IGV tracks for Gata2 from Chip-seq data. Con, C: control, Met, T: metformin. **D** Verification of Gata2 expression by qRT-PCR. (E) Verification of Gata2 expression by western blotting. Uncropped full-length gel blots can be found in Additional file 5: Fig. S5C. **F** Analysis of FGSCs with Gata2 interference by CCK-8 assays (Scale bars: 20 μm). **G** EdU assay of FGSCs with Gata2 interference. **H** EdU assay of Gata2-knockdown FGSCs with metformin treatment. All data are presented as means ± SD of three biological replicates. *p < 0.05, **p < 0.01 compared with the control by two-tailed Student’s t-test. Con: control, Met: metformin

After analysis of H2BK5bhb ChIP-seq and RNA-seq data, we chose three different genes that simultaneously had an increase of H2BK5bhb in the promoter region and upregulation of gene expression. ChIP-qPCR assays confirmed that H3BK5bhb was enriched in three promoter regions of target genes (Fig. 5A). Among these candidate genes, the transcription factor Gata2 has been reported to act as a self-renewal maintenance factor in hematopoietic stem cells [50, 51], which might be a target gene for metformin to regulate FGSC development.

### Metformin promotes FGSC proliferation by modifying histone H2BK5bhb of Gata2

To confirm activation of Gata2 transcription by H2BK5bhb, we analyzed the Gata2 promoter region signal in ChIP-seq data. The results showed that metformin treatment increased signal enrichment in the Gata2 gene transcription start site (TSS) ± 5 kb region, and an IGV diagram indicated that metformin treatment led to significant signal enrichment in the whole Gata2 gene sequence region including the promoter (Fig. 5B, C). Furthermore, qRT-PCR and western blotting confirmed that mRNA and protein expression of Gata2 was increased after metformin treatment (Fig. 5D, E). Uncropped full-length gel blots can be found in Additional file 5: Fig. S5C. To reveal the role of Gata2 in FGSC development, we knocked down Gata2 expression in FGSCs (Additional file 4: Fig. S4, Additional file 9: Table S4). Uncropped full-length gel blots can be found in Additional file 5: Fig. S5D. Using CCK-8 assays, we assessed the effect of Gata2 knockdown on FGSC viability. The results showed that FGSC viability was decreased significantly compared with the control group after 24 h of culture (Fig. 5F). To determine the effect of Gata2 on FGSC proliferation, we performed an EdU assay. The results showed that the FGSC proliferation rate in the Gata2-knockdown group was reduced significantly (Fig. 5G). Similarly, to further confirm that metformin promoted the proliferation of FGSC via Gata2, EdU assays were performed in Gata2-knockdown FGSC after metformin treatment. The results showed that the proliferation rate of metformin-treated FGSC (sh-NC-Met) was significantly higher than control group (sh-NC) (Fig. 5H). Furthermore, the proliferation rate of Gata2-knockdown FGSC (sh-Gata2) was significantly lower than that of control group (Fig. 5H). There were no significant differences between Gata2-knockdown FGSC and Gata2-knockdown FGSC treated with metformin (sh-Gata2-Met) (Fig. 5H). Taken together, these data indicated that metformin promoted FGSC proliferation by modifying histone H2BK5bhb of Gata2.

## Discussion

Infertility has become a serious threat to human health as cancer, cardiovascular and cerebrovascular diseases. Metformin, a synthetic and safe anti-type II diabetes drug, has been shown to improve oxidative stress-induced cell damage, inhibit inflammatory responses, regulate the autophagy pathway, and delay aging [3]. Additionally, metformin has a reproductive regulatory effect on follicular development and is used to treat pregnancy-induced hyperglycemia in the menstrual cycle of PCOS patients [12, 15]. In this study, we conducted in vivo experiments to verify the regulatory effect of metformin on ovarian functions in mice. Our findings show that after one week of treatment, the progesterone level in 12-week-old mice significantly increased, indicating a luteinizing effect, while 28-week-old mice showed promoted follicular development and a significant increase in the serum level of estradiol. Three weeks after treatment, both 12- and 28-week-old mice showed an increase in E2 and a decrease in progesterone. In terms of follicular development, at 1 week after treatment, the number of corpus luteum was increased significantly in 12-week-old mice, and the numbers of primordial and primary follicles were significantly increased in 28-week-old mice, which were consistent with the results of hormone secretion. After 3 weeks of metformin treatment, secondary and antral follicles were significantly increased in the ovaries of mice of different ages. This increase in secondary and antral follicles may be due to the primordial and primary follicles affected by metformin for one week, continuing to develop into secondary and antral follicles. Female germ stem cells (FGSCs) play a crucial role in improving female fertility by providing female gametes for offspring development, which is another important aspect of improving female fertility [23]. Our in vivo analysis of the location and numbers of FGSCs in mouse ovaries showed that metformin induced a consistent increase in the number of FGSCs in mice at 12 and 28 weeks of age, and the effect weakened over time. These results showed that metformin regulated ovarian functions and increased the number of ovarian FGSCs. In vitro experiments were conducted to further study the effect of metformin on FGSC development, which showed that metformin promoted FGSC proliferation, consistent with a previous study [20]. Thus, we identified a proliferation-inducing effect of metformin on FGSCs in vitro.

Kbhb is a novel histone acylation modification that has been identified in various species, including mice and humans [42]. As a new histone marker, 44 core histone hydroxybutyrylation sites were identified in mice and humans, including H4K8, H4K12, H3K4, H3K9, and H3K56 [42]. During low-carbon nutrition, an increase in β-hydroxybutyrylation in the promoter region has been associated with upregulation of active genes in the starvation-responsive pathway [42]. In our study, we found that metformin changed the Kbhb modification of FGSCs as represented by a downregulation trend in the approximately 15-kD histone core subunit region. Combined with Kbhb modification sequencing analysis, we observed that the change in the histone Kbhb modification after metformin treatment occurred in H2A and H2B subunits, which was different from histone core subunits H3 and H4 as reported in most other studies. Furthermore, sites with significant modification changes, including H2AK95, H2BK120, H2BK108, H2BK5, and H2BK46, were different from the main research site of histone Kbhb modification, H3K9bhb. Our study revealed novel characteristics of the histone Kbhb modification induced by metformin in FGSCs. However, the mechanism of metformin-regulating histone Kbhb modification in FGSCs and its involvement in the β-hydroxybutyric acid metabolic pathway or the activity of β-hydroxybutyryl-CoA metabolic enzymes requires further exploration.

Gata2 is a DNA transcriptional regulator with a zinc finger structure belonging to the Gata family. It was first discovered in hematopoietic stem cells and is essential for maintaining cell numbers, self-renewal, and hematopoiesis in hematopoietic stem cells and pluripotent progenitor cells [50–52]. Additionally, Gata2 regulates many genes essential for embryonic development, lymphatic system formation, and self-renewal of other tissue stem cells [53]. Our combined analysis by H2BK5bhb Chip-seq and transcriptome RNA-seq indicated that both the H2BK5bhb modification in the Gata2 promoter region and its gene expression were upregulated after metformin treatment. Subsequent interference experiments showed that Gata2 played a crucial role in maintaining the viability and proliferation of FGSCs. However, the interactions of Gata2 with other trans-acting factors or histone modification sites, transcriptional initiation regulation of downstream genes, and the signal pathways involved require further investigation.

## Conclusions

**Fig. 6:**
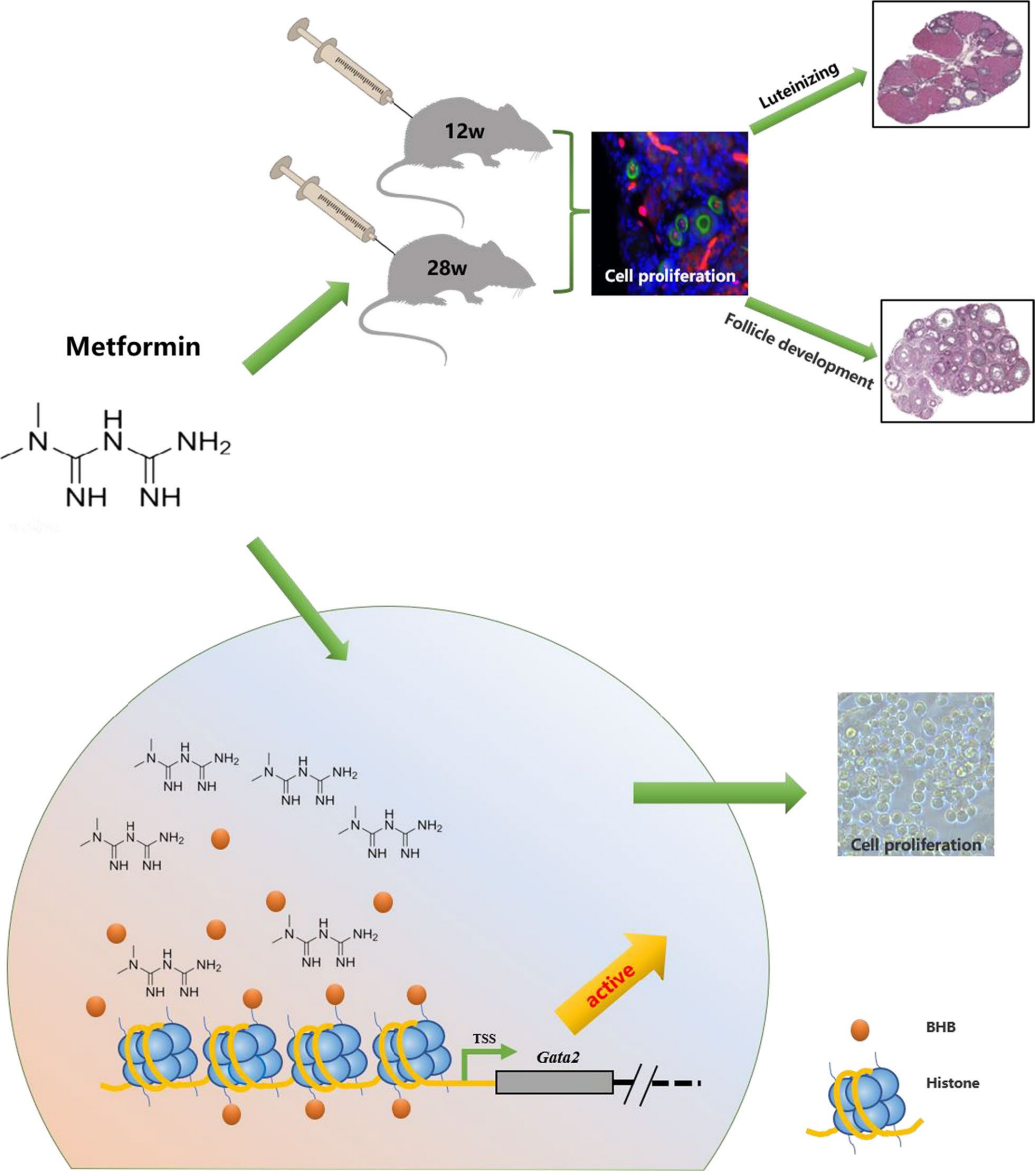
Schematic diagram of metformin-regulating mouse ovarian functions and promoting FGSC proliferation. Metformin increased the number of FGSCs in mouse ovaries, promoted follicular development and luteogenesis, and promoted FGSC proliferation by upregulating Gata2 with histone β-hydroxybutyrylation

This study demonstrated that metformin promotes the development of mouse ovarian FGSCs and follicles, affects the Kbhb of an FGSC line, and upregulates Gata2 expression by increasing the histone H2BK5bhb modification of the Gata2 promoter region, ultimately promoting FGSC proliferation (as shown in Fig. 6). These findings regarding the proliferation-promoting effect of metformin on FGSCs and the identification of histone H2BK5bhb modification targets support studies on fate determination and epigenetic modification regulation of FGSCs, providing a theoretical basis for the clinical application of metformin in female fertility maintenance.

## Supplementary Information

**Additional file 1**. **Figure S1**. Characteristics of the FGSC line in vitro.Identification of mRNA expression of FGSC-related markers by RT-PCR. Uncropped full-length agarose gels can be found in Fig. S5E-G.Top: Cell morphology of FGSCs in bright field. Bottom: Immunofluorescence staining of Mvh in FGSCs

**Additional file 2**. **Figure S2**. Histone H2BK5bhb Chip-seq quality control chart. The 300–500 bp fragment had continuous enrichment, which was the main fragment region selected for library construction, high-throughput sequencing, and data analysis, which met the requirements of chromatin fragment preparation

**Additional file 3**. **Figure S3**. Mass fraction distribution of the RNA-seq base position. Sequencing data revealed the position of the specific sequencing signal in each replicated group by FastQC.

**Additional file 4**. **Figure S4**. Verification of Gata2 interference efficiency.Gata2-lentivirus infection efficiency was determined by fluorescence microscopy.Interference efficiency of mRNA levels was evaluated by qRT-PCR.Left: Interference efficiency of the protein level was validated by western blotting. Right: Statistical analysis of western blots. Uncropped full-length gel blots can be found in Fig. S5D. All data are presented as means ± SD of three biological replicates. **p < 0.01 compared with the control by one-way ANOVA and the multiple comparisons test.

**Additional file 5**. **Figure S5**. Corresponding uncropped full-length gels and blot.Changes of Kbhb in the histone region of FGSCs treated with metformin as determined by western blotting.Western blot validation of histone H2BK5bhb modification.Verification of Gata2 expression by western blotting.Interference efficiency of the protein level was validated by western blotting.,,Identification of mRNA expression of FGSC-related markers by RT-PCR.

**Additional file 6**. **Table S1**. Sequence of primers used in regular RT-PCR

**Additional file 7**. **Table S2**. Sequence of primers used in Quantitative RT-PCR

**Additional file 8**. **Table S3**. Sequence of primers used in ChIP-qPCR

**Additional file 9**. **Table S4**. Gata2 shRNA sequences

## Data Availability

The RNA-seq, ChIP-seq data have been submitted to the NCBI Gene Expression Omnibus database under accession number GSE217784 and can be accessed by entering the GSE accession number (GSE217784) at the following URL (https://www.ncbi.nlm.nih.gov/geo/query/acc.cgi?acc). The mass spectrometry proteomics data have been deposited to the ProteomeXchange Consortium (http://proteomecentral.proteomexchange.org) with the dataset identifier PXD038090.

## Abbreviation

FGSCs: Female germline stem cells
bhb: β-Hydroxybutyrylation
Kbhb: Histone Lys β-hydroxybutyrylation
H2BK5bhb: Histone H2B Lys5 β-hydroxybutyrylation
Gata2: Gata-binding protein 2
PCOS: Polycystic ovarian syndrome
HE: Hematoxylin and eosin
STO: SIM mouse embryo-derived thioguanine and ouabain-resistant
CCK-8: Cell counting kit-8
EDU: 5-Ethynyl-2′-deoxyuridine
DAPI: 4′,6-d-Iamidino-2-phenylindole
ChIP: Chromatin immunoprecipitation
GO: Gene ontology
KEGG: Kyoto encyclopedia of genes and genomes
TSS: Transcription start site
