# Supplementary figures and images for: Metformin promotes female germline stem cell proliferation by upregulating Gata-binding protein 2 with histone β-hydroxybutyrylation
Source: Stem Cell Res Ther. 2023 May 26;14:144. doi: 10.1186/s13287-023-03360-1 (PMC10214601; doi:10.1186/s13287-023-03360-1)

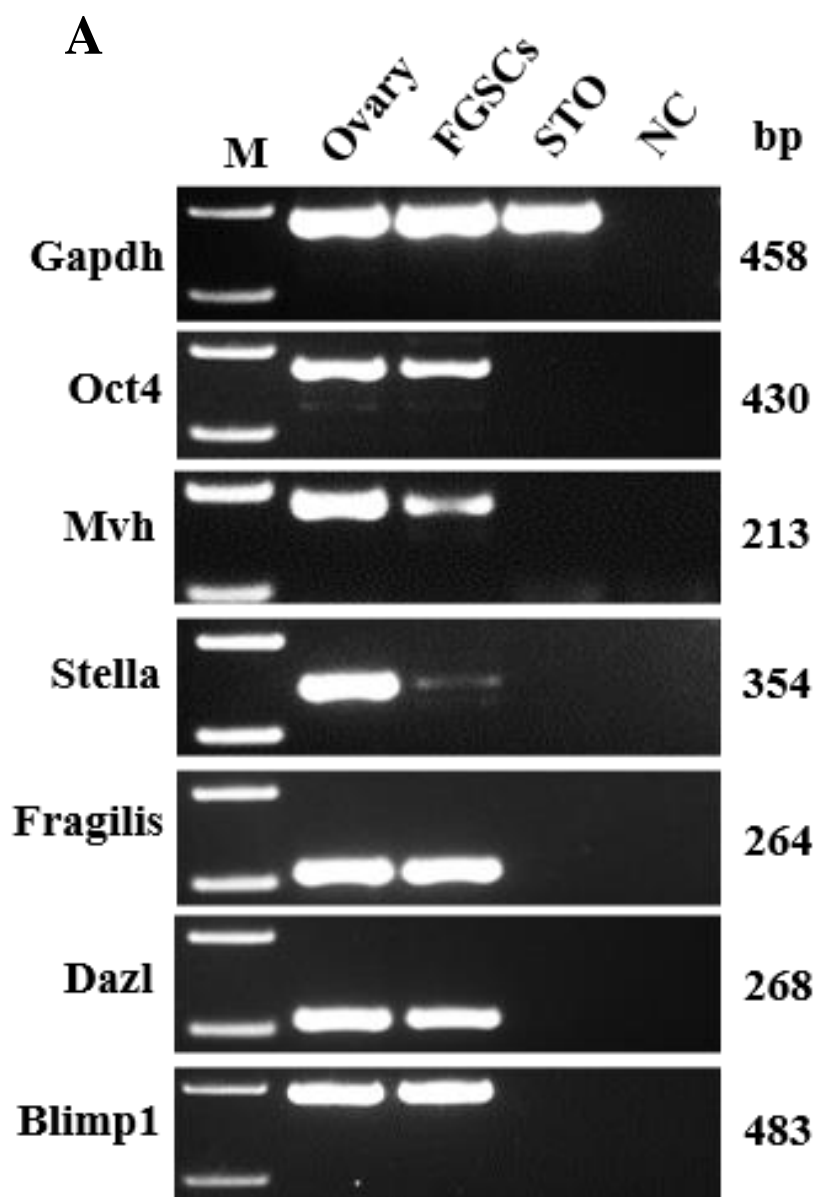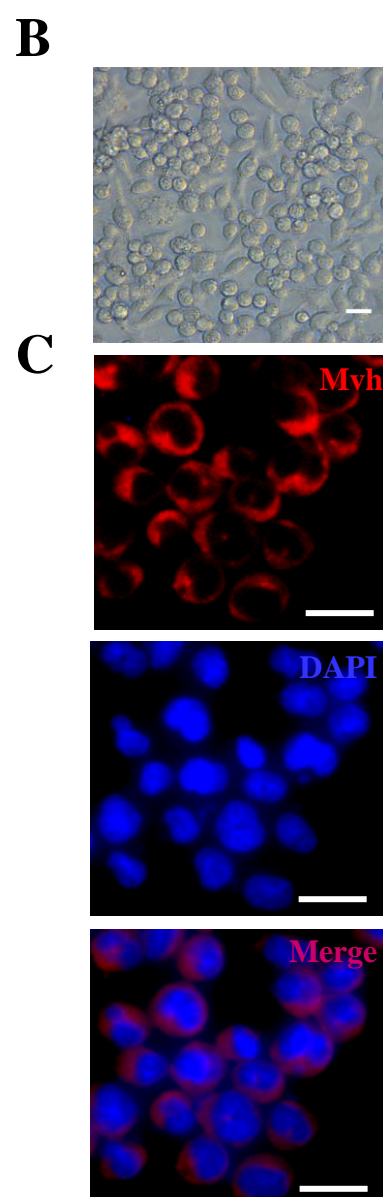

Supplement: Supplementary file 1 — Additional file 1. Figure S1. Characteristics of the FGSC line in vitro.Identification of mRNA expression of FGSC-related markers by RT-PCR. Uncropped full-length agarose gels can be found in Fig. S5E-G.Top: Cell morphology of FGSCs in bright field. Bottom: Immunofluorescence staining of Mvh in FGSCs [file 13287_2023_3360_MOESM1_ESM.pdf]

**Sample intensity**

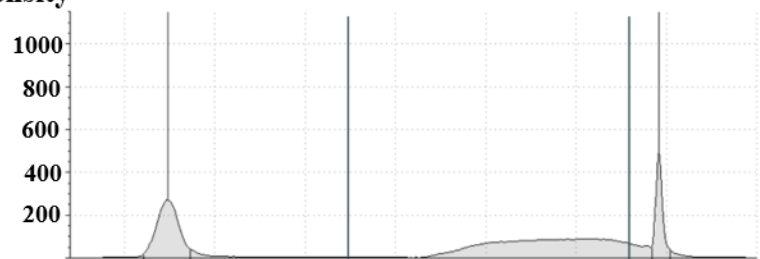

**C-input**

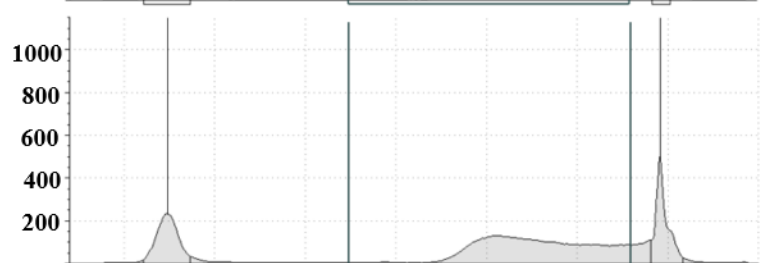

**C-ip**

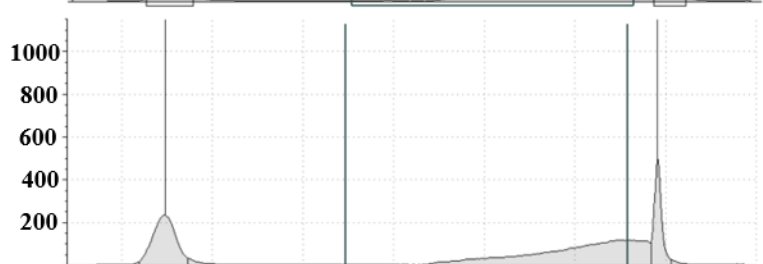

**T-input**

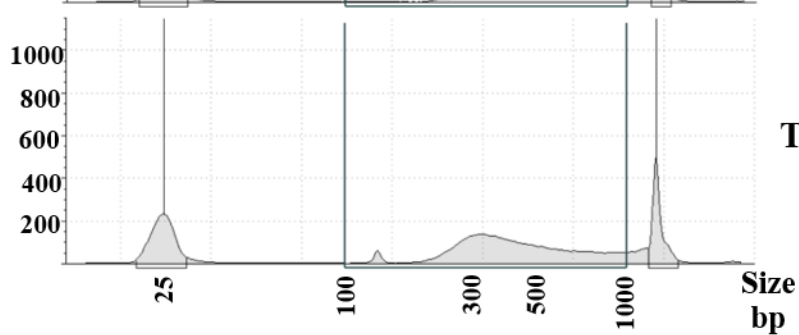

**T-ip**

**Size  
bp**

Supplement: Supplementary file 2 — Additional file 2. Figure S2. Histone H2BK5bhb Chip-seq quality control chart. The 300–500 bp fragment had continuous enrichment, which was the main fragment region selected for library construction, high-throughput sequencing, and data analysis, which met the requirements of chromatin fragment preparation [file 13287_2023_3360_MOESM2_ESM.pdf]

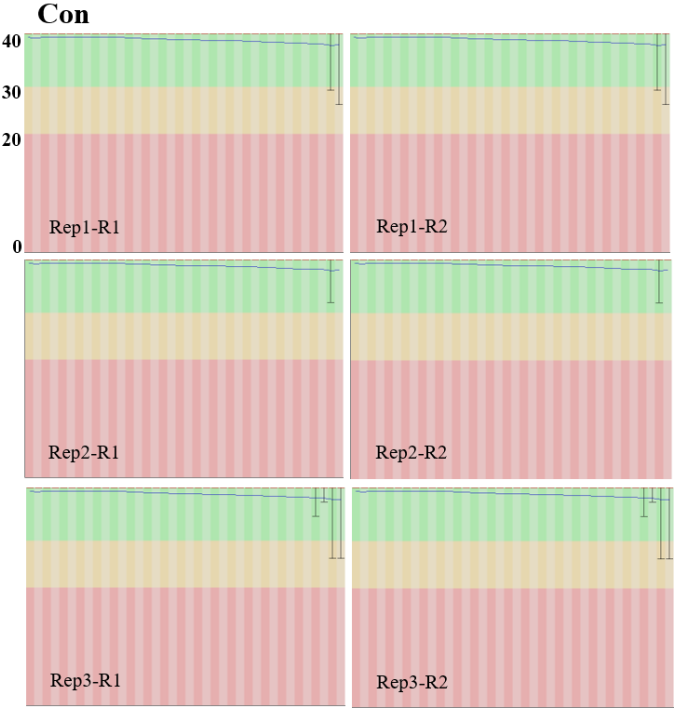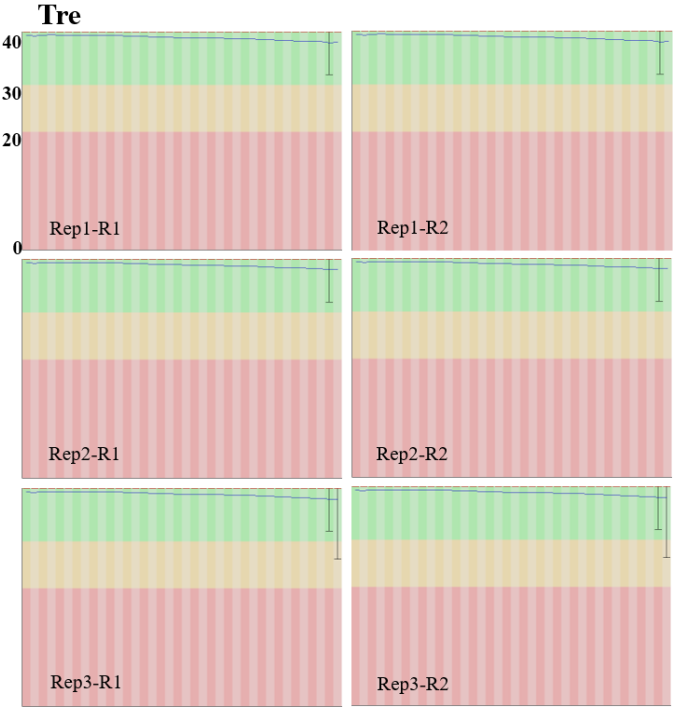

Supplement: Supplementary file 3 — Additional file 3. Figure S3. Mass fraction distribution of the RNA-seq base position. Sequencing data revealed the position of the specific sequencing signal in each replicated group by FastQC. [file 13287_2023_3360_MOESM3_ESM.pdf]

**A**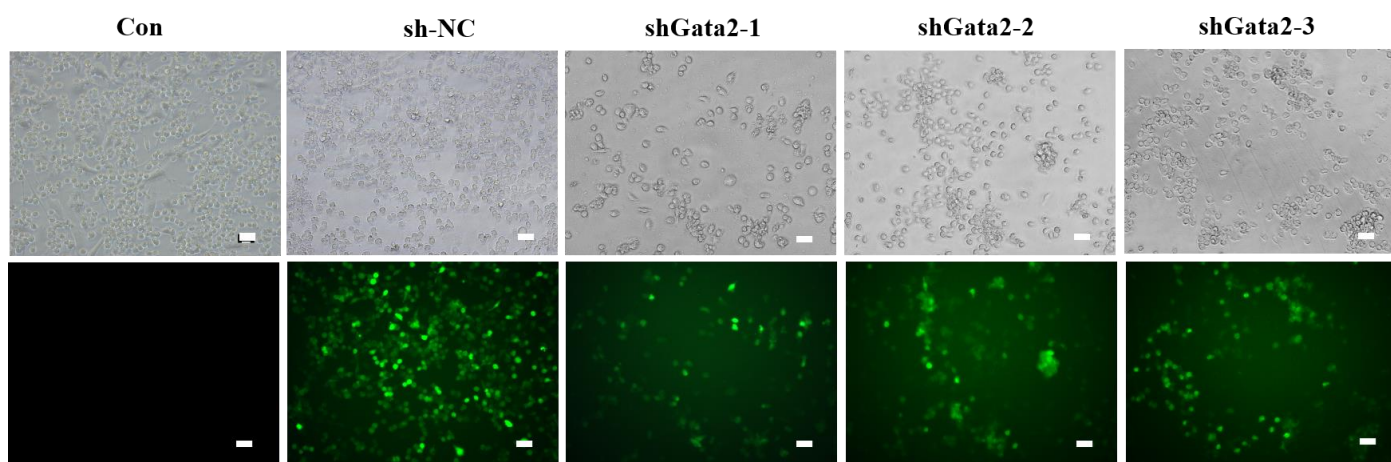**B**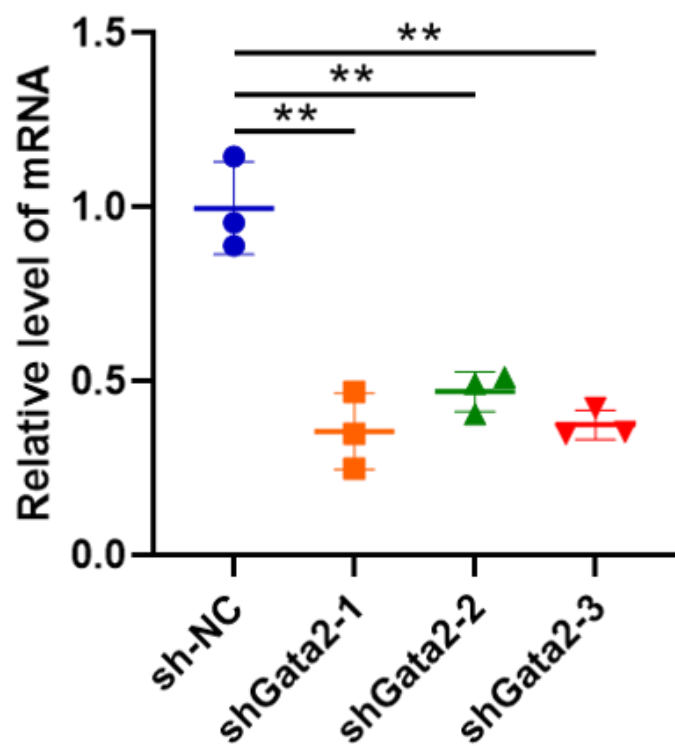**C**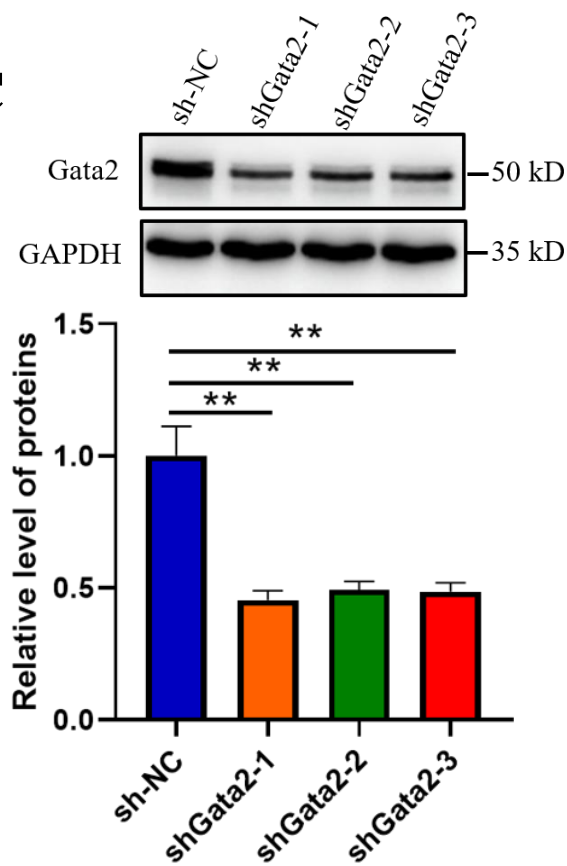

Supplement: Supplementary file 4 — Additional file 4. Figure S4. Verification of Gata2 interference efficiency.Gata2-lentivirus infection efficiency was determined by fluorescence microscopy.Interference efficiency of mRNA levels was evaluated by qRT-PCR.Left: Interference efficiency of the protein level was validated by western blotting. Right: Statistical analysis of western blots. Uncropped full-length gel blots can be found in Fig. S5D. All data are presented as means ± SD of three biological replicates. **p < 0.01 compared with the control by one-way ANOVA and the multiple comparisons test. [file 13287_2023_3360_MOESM4_ESM.pdf]

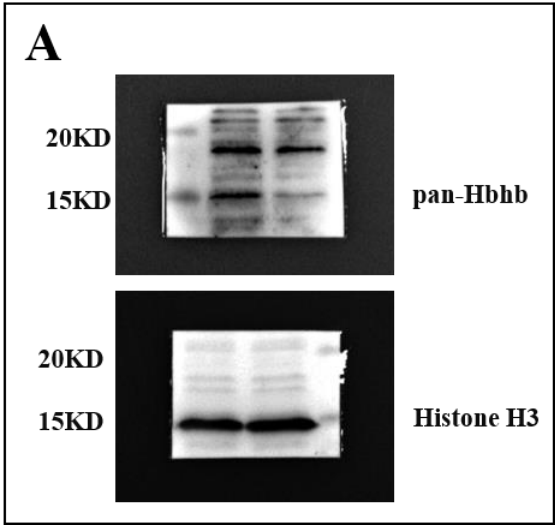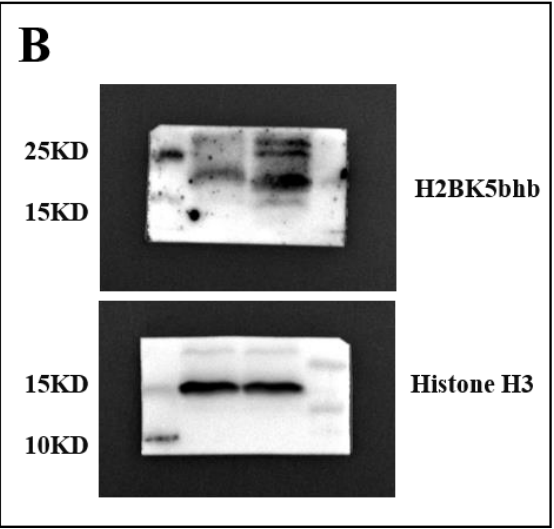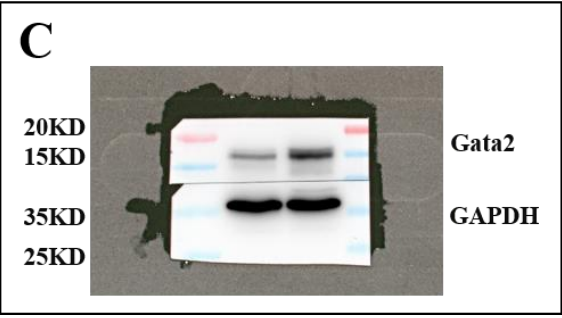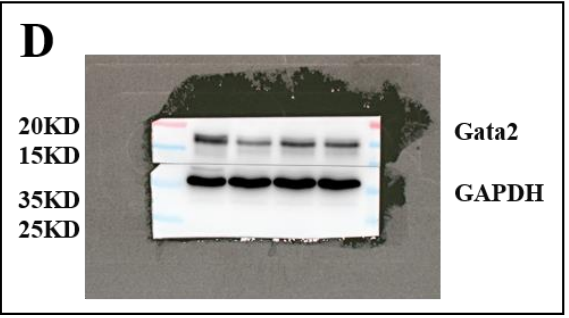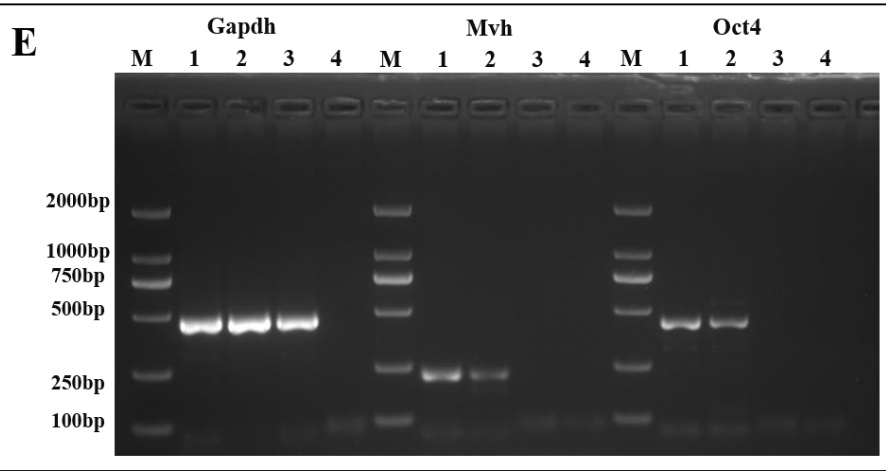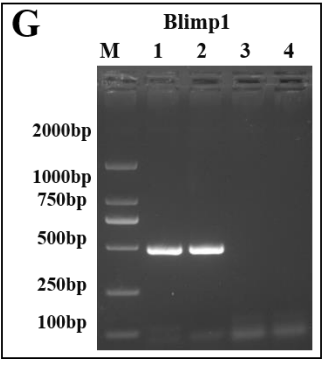

M: maker DL2000  
1: Ovary  
2: FGSCs  
3: STOs  
4: Negative control

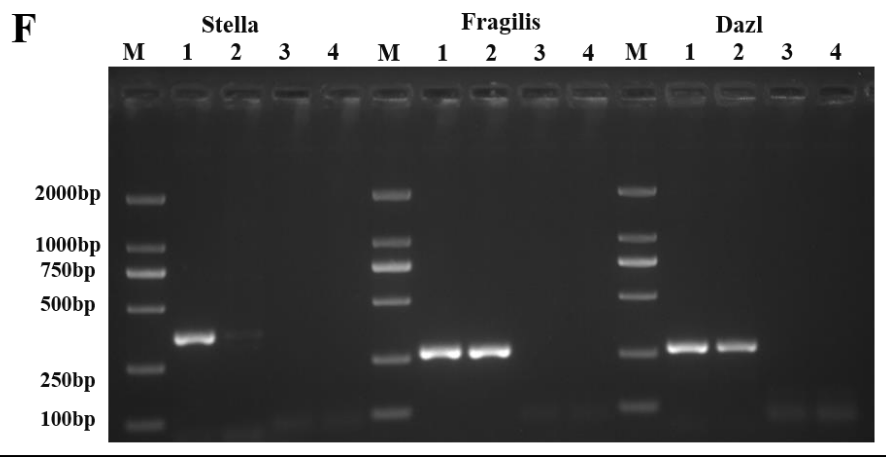

Supplement: Supplementary file 5 — Additional file 5. Figure S5. Corresponding uncropped full-length gels and blot.Changes of Kbhb in the histone region of FGSCs treated with metformin as determined by western blotting.Western blot validation of histone H2BK5bhb modification.Verification of Gata2 expression by western blotting.Interference efficiency of the protein level was validated by western blotting.,,Identification of mRNA expression of FGSC-related markers by RT-PCR. [file 13287_2023_3360_MOESM5_ESM.pdf]
